# The prognosis significance and application value of peritoneal elastic lamina invasion in colon cancer

**DOI:** 10.1371/journal.pone.0194804

**Published:** 2018-04-09

**Authors:** Jun Lu, Xiumei Hu, Yutong Meng, Hongying Zhao, Qing Cao, Mulan Jin

**Affiliations:** Department of Pathology, Beijing Chaoyang Hospital, Capital University, Beijing, China; National Health Research Institutes, TAIWAN

## Abstract

**Objectives:**

The aims of this study were to evaluate the associations between peritoneal elastic lamina invasion (ELI) and the clinicopathological prognostic factors of colon cancer, to evaluate the feasibility of ELI with use of an elastic stain to help diagnose serosal invasion of colon cancer in routine practice, so as to help us to provide a more accurate estimate for prognosis and stage of patients and a marker for postoperative treatment.

**Methods:**

254 cases with colon cancer were included in the study. According to the presence of elastic lamina (EL) and elastic lamina invasion (ELI), all cases were divided into four groups: pT3 EL negative (pT3 EL (-)), pT3 ELI positive (pT3 ELI (+)), pT3 ELI negative (pT3 ELI (-)) and pT4a. Statistical analysis was used to analyze the relationship between elastic lamina invasion and other established adverse histologic features.

**Results:**

The EL and ELI positive rates were 81.5% and 42.1% respectively. There were significant differences in mph node metastasis, venous invasion and tumor buds between pT3 ELI (-) and pT3 ELI (+), pT3 ELI (-) and pT4a. There was no significant difference in same factors between pT3 ELI (+) and pT4a. In pT3 stage, there were significant differences in lymph node metastasis, perineural invasion and tumor buds between EL (-) and ELI (+). There were no significant differences in same factors between EL (-) and ELI (-). EL was detected less frequently in right-sided tumors compared with left-sided tumors.

**Conclusions:**

ELI might be the prognostic factors of colon cancer with II stage and might be the marker of postoperative adjuvant chemotherapy. Patients with pT3 ELI (+) might have similar prognosis to patients with pT4a. For patients with pT3 colon cancer, EL(-) might have similar prognosis as ELI (-) and might take the same therapy. In addition, the right half colon EL positive rate was lower than the left colon. Elastic staining might be a useful tool to help determine the invasive depth and stage of colon cancer.

## Introduction

Peritoneal involvement was an important adverse prognostic factor in colorectal cancer (CRC). Serosal invasion could distinguish between T3 and T4a stage colon cancer and may prompt consideration of adjuvant chemotherapy in stage II disease. The common accepted definition of serosal invasion is surface cells of layer had been damaged by the tumor cells [1–2]. Actually, it was very difficult to accurately judge the serosal invasion only by hematoxylin and eosin (H&E) stain.

The methods could help to confirm serosal invasion including cytological examination and Immunohistochemistry. It should be noted that the presence of malignant cells in peritoneal fluid could be a consequence of tumor metastases to lymph nodes or other sites and therefore would not necessarily be the result of direct trans-serosal spread by the primary tumor. As for Immunohistochemistry, because of damage of the surface of serosa caused by the fibrous and inflammatory, the diagnostic positive rate of this method is not high. Accordingly, these techniques were not universally applied in routine histopathological analysis.

Peritoneal elastic lamina (PEL) comprised a relatively delicate layer of elastic fibers that lied just deep to the mesothelium. Some report showed that elastic lamina (EL) was the part of the serosa [3]. Elastic fiber had strong resistance of damage, was generally not easy to break. Shinto et al. first put forward the application of elastic staining to screen high-risk group of patients with pT3 CRC [4]. As a sign of pleural invasion, elastic lamina invasion (ELI) had been applied to the clinical diagnosis and treatment of lung cancer [5]. Therefore, PEL, probably as a marker of serosal invasion for CRC, would be used for auxiliary diagnosis of tumor invasion depth and a marker of postoperative chemotherapy for patients with stage II colon cancer.

Herein, we evaluated the associations between peritoneal ELI and the clinicopathological prognostic factors of colon cancer. We would like to highlight the feasibility of ELI with use of an elastic stain to help diagnosing serosal invasion of colon cancer in routine practice, so as to help us to provide a more accurate estimate for prognosis and stage of patients and a marker for postoperative treatment.

## Materials and methods

### Clinical materials

254 cases for the first diagnosis of a colonic cancer at Beijing chaoyang hospital affiliated to the capital University from October 2003 to October 2009 were included into the study. This study was retrospective and the data were fully anonymized before we had access to them. All specimens were with the approval of Beijing chaoyang hospital affiliated to the capital University ethics committee (NO.2015-KE-41). Inclusion criteria: (1) specimens treated for colon adenocarcinoma, including ascending colon, transverse colon, descending colon and sigmoid colon; (2) stage: pT3 and pT4a. (3) At least 1 piece HE slices with serosa or highly suspicious remaining serosa. Eliminate cases: (1) recurrent colon cancer; (2) multiple primary colon cancer, familial adenomatous polyp patient; (3) preoperative neoadjuvant chemotherapy.

### Methods

#### The clinicalpathology data

Two experienced gastrointestinal GI) pathologist reviewed all cases at the same time. The clinical pathological features included stage, differentiation, tumor bud, nerve and venous invasion and lymph node metastasis. If a case was controversial, they would show the case to the third GI pathologist and attain consensus. All relevant data are within the paper. Stage: (1) pT3 and pT4a were performed according to the TNM staging system of colorectal cancer. (2) Differentiation: All cases were divided into three groups: well-differentiated, moderately differentiated and poorly differentiated adenocarcinoma. Mucous adenocarcinoma and signet ring cell carcinoma was taken as poorly differentiated adenocarcinoma. (3) Tumor bud: Tumor buds were defined as the presence of single tumor cells or a clusters of up to five tumor cells at the invasive tumor front. Referencing to CRC treatment guidelines in Japan (2010) tumor bud classification standard, tumor bud could be divided into 3 level, namely 0 to 4 tumor bud for level 1, 5–9 tumor bud of level 2, 10 buds for level 3 [6].

#### H&E and elastic lamina staining and staining analysis

In each case, at least one wax block with serosa or highly suspected serosa was selected for elastic lamina and hematoxylin and eosin (H&E) staining. The slices were cut into 4mm sections and stained with H&E and elastica stain for histologic evaluation. Ventana SYMPHONY H&E system and Ventana NexES automatic special staining machine (Roche diagnosis) were used for H&E and elastic fiber staining. Elastic fiber staining protocol were followed as the detailed instructions given by the machine: paraffin block, with 4 microns section at 60 ° C oven for 20 min; Dimethyl benzene dewaxing, hydration, gradient alcohol distilled water flushing; Elastic fiber staining NexES automatic special staining machine; Run immediately after the end would be organized, quick wipe surface residual reagents with pre-configured rinses fast dipping washed three times; Gradient alcohol dehydration, transparent, neutral gum sealing piece. PEL invasion was evaluated using the criteria proposed by Grin A [7]: (1) the tumor could be clearly seen exceeded the peritoneal elastic lamina;(2) tumor was present beyond the level traced between residual elastic present on either side of the tumor in cases where a fibro-inflammatory reaction obscured the elastic lamina in the region of the tumor. (3) for mucinous tumors, ELI was scored as positive only if cellular mucin breached the elastic lamina; (4) if the elastic fiber staining showed a repeated elastic lamina structure, only tumor invasion more than the most outer layer elastic lamina was diagnosed with ELI.(5) in one or more slices in the above situation, namely, the cases can be diagnosed with ELI.(6) the case showing the elastic lamina by microscope was defined as positive elastic lamina (EL(+)), no elastic plate display was defined as the negative elastic lamina (EL (-)). Depending on the depth of tumor invasion, tumors that exceed the elastic lamina were defined as positive ELI (+) and tumors that did not exceed the elastic lamina was defined as ELI (-).

#### Statistical analysis

Descriptive statistics, including medians and percentages were calculated, chi-square analysis and spearman correlation analyses were applied to show the relationship between the clinical pathological characteristics and the PEL invasion. p<0.05 was considered to indicate a significant difference in all analyses. All analyses were carried out using SPSS 16.0 software [IBM Corporation].

## Results

### Demographics and pathology

The average age at diagnosis was 66.2 years (28-88y). There were 147 males and 107 females, with a ratio of 1.37: 1. There were 124 cases of right colon and 130 cases of left colon. There were 11 cases of well-differentiated adenocarcinomas, 204cases of moderately differentiated adenocarcinoma and 39 cases of poorly differentiated adenocarcinoma. There were 225 patients in pT3 stage and 29 patients in pT4a period. In 114 patients (44.8%) lymph node metastases were found. 61 cases confirmed by elastic fiber staining could be seen infiltrated veins and 55 cases could be seen visible nerve invasion. There were 127 cases of Tumor bud grade 1, 52 cases of grade 2 and 75 cases of grade 3.

### Positive rate of EL and ELI

**Fig 1:**
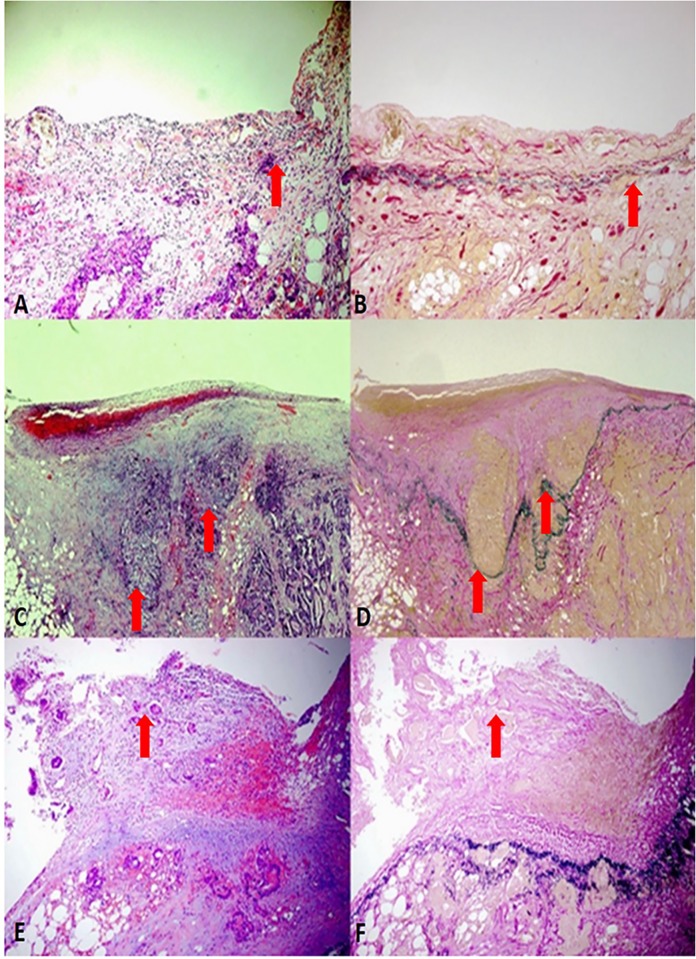
A:Tumor cells approached the serosa, as shown by the red arrow (H&E, 200 x) .B: The deepest tumor cells approached PEL but did not exceed PEL highlighted with an elastic stain. As shown by the red arrow (Elastic fiber staining, 200 x) . C: Tumor cells invaded the serosa, accompanied by significant inflammatory response, fibrous tissue and angiogenesis hyperplasia. as shown by the red arrow (H&E, 100 x) .D: Invasion through PEL highlighted with an elastic stain, as shown by the red arrow (Elastic fiber staining, 100 x) . E: Tumor cells appeared on the serosal surface, accompanied by inflammatory responses and erosion, as shown by the red arrow (H&E, 100 x) . F: Invasion through PEL highlighted with an elastic stain, as shown by the red arrow (Elastic fiber staining, 100 x).

A total of 822 blocks of 254 patients was taken HE and elastic staining. The average blocks for elastic fibers staining for per case was 3.24 (2–5). The elastic lamina was identified in 207 cases (EL (+), 81.5%) using elastic fibers stain, but could not be identified in 47 cases (EL (-), 18.5%). ELI (ELI+) was identified in 107 cases (42.1%). Representative pictures of elastic lamina assessment are shown in Fig 1. According to pT stage, based on the presence of elastic lamina and the elastic lamina invasion, all the cases were divided into stage pT3 EL (-) (18.5%), pT3 ELI (+) (30.7%), pT3 ELI (-) (39.4%) and pT4a (11.4%).

### pT3 ELI (+), pT3 ELI (-) and pT4a

There were no significant differences in age, gender, tumor location and differentiation between three groups (P > 0.05), as shown in Table 1. There were significant differences in tumor buds, venous invasion, lymph node metastasis and neural invasion between three groups (P < 0.05). There were significant difference and positive correlation (P < 0.05, R value: 0.272, 0.148, 0.229, 0.474) in lymph node metastasis, venous invasion, nerve invasion and tumor buds between pT3 ELI (-) and pT3 ELI (+). There were significant difference and positive correlation (P < 0.05, R value: 0.248, 0.257, 0.524) in lymph node metastasis, venous invasion and tumor buds between pT3 ELI (-) and pT4a. However, there were no significant differences in lymph node metastasis, venous invasion, nerve invasion, and degree of tumor buds between pT3 ELI (+) and pT4a, as shown in Table 2.

**Table 1:** Clinicopathological features of patients in pT3 ELI (+),pT3 ELI (-) and pT4a.

		T3 EIL (-)(%)	T3 ELI (+) (%)	T4a (%)	P value
Age(average±standard deviation)		67.9±10.9	64.8±12.6	65.3±12.8	0.486
gender					
	female	45 (21.7)	37 (17.9)	12 (5.8)	0.850
	male	55 (26.6)	41 (19.8)	17 (8.2)	
location					
	right colon	49 (23.7)	29 (14.0)	12 (5.89)	0.632
	left colon	51 (24.6)	49 (23.7)	17 (8.2)	
differentiation					
	high	7 (3.4)	2 (1.0)	0 (0)	0.284
	moderate	77 (37.2)	65 (31.4)	22 (10.6)	
	low	16 (7.7)	11 (5.3)	7 (3.4)	
Lymph node metastasis					
	negative	67 (32.4)	31 (15.0)	11 (5.3)	0.000
	positive	33 (15.9)	47 (22.7)	18 (8.7)	
Venous invasion					
	negative	84 (40.6)	56 (27.1)	17 (8.2)	0.011
	positive	16 (7.7)	22 (10.6)	12 (5.8)	
Nerve invasion					
	negative	84 (40.6)	50 (24.2)	22 (10.6)	0.009
	positive	16 (7.7)	28 (13.5)	7 (3.4)	
Tumor bud					
	Level 1	68 (32.9)	24 (11.6)	7 (3.4)	0.000
	Level 2	24 (11.6)	13 (6.3)	3 (1.4)	
	Level 3	8 (3.9)	41 (19.8)	19 (9.2)	

**Table 2:** Clinicopathological characteristics of correlation of patients in pT3 ELI (+),pT3 ELI (-) and pT4a.

		T3 ELI (-) VS T3 ELI (+)	T3 ELI (-) VS T4a	T3ELI (+) VS T4a
Lymph node metastasis				
	P	0.000<0.05	0.005<0.05	0.864
	R	0.272	0.248	0.016
Venous invasion				
	P	0.049<0.05	0.004<0.05	0.193
	R	0.148	0.257	0.126
Nerve invasion				
	P	0.002<0.05	0.313	0.249
	R	0.229	0.089	0.111
Tumor bud				
	P	0.000<0.05	0.000<0.05	0.468
	R	0.474	0.524	0.099

### pT3 EL (-),pT3 ELI (+) and pT3 ELI (-)

There were 47 cases shown no PEL, accounted for 18.5% of all cases and 20.9% of pT3 stage. All cases were confirmed by repeating stain for all blocks of tumor and confirmed by two gastrcintestal pathologist. There were significant differences in lymph node metastasis, nerve invasion, tumor buds and tumor location between three groups, as shown in Table 3. There was no significant difference (P > 0.05) in lymph node metastasis, nerve invasion and tumor buds between the EL (-) and ELI (-), as shown in Table 4. However between the group of EL (-) and ELI (+), there were significant difference and positive correlation (P < 0.05, R value: 0.254, 0.304 and 0.360).

**Table 3:** Clinicopathological characteristics of patients in pT3 EL (-),pT3 ELI (+) and pT3 ELI (-).

		T3 EL (-) (%)	T3 ELI (-) (%)	T3ELI (+) (%)	P value
Age(average±standard deviation)		67.7±9.7	64.8±12.0	67.5±12.2	0.430
gender					
	female	13 (5.8)	45 (19.9)	37 (16.4)	0.072
	male	34 (15.1)	55 (24.4)	41 (18.2)	
location					
	right colon	34 (5.1)	49 (21.8)	29 (12.9)	0.001
	left colon	13 (5.8)	51 (22.7)	49 (21.8)	
differentiation					
	high	2 (0.9)	7 (3.1)	2 (0.9)	0.589
	mederate	40 (17.7)	77 (34.1)	65 (28.9)	
	low	5 (2.2)	16 (7.1)	11 (4.9)	
Lymph node metastasis					
	negative	31 (13.7)	67 (29.6)	31 (14.2)	0.001
	positive	16 (7.1)	33 (14.6)	47 (20.8)	
Venous invasion					
	negative	36 (16.0)	84 (37.3)	56 (24.9)	0.141
	positive	11 (4.9)	16 (7.1)	22 (9.8)	
Nerve invasion					
	negative	43 (19.1)	84 (37.3)	50 (22.2)	0.000
	positive	4 (1.8)	16 (7.1)	28 (12.4)	
Tumor bud					
	Level 1	28 (12.4)	68 (30.2)	24 (10.7)	0.000
	Level 2	12 (5.3)	24 (10.7)	13 (5.8)	
	Level 3	7 (3.1)	8 (3.6)	41 (18.2)	

**Table 4:** Clinicopathological characteristics of correlation of patients in pT3 EL (-),pT3 ELI (+) and pT3 ELI (-).

		EL (-) VS ELI (-)	EL (-) VS ELI (+)
Lymph node metastasis			
	P	0.900	0.004<0.05
	R	0.010	0.254
Nerve invasion			
	P	0.217	0.001<0.05
	R	0.102	0.304
Tumor bud			
	P	0.393	0.000<0.05
	R	0.106	0.360

### The relationship between tumor site and EL

There were significant differences between pT3 EL (-),pT3 ELI (-) and pT3 ELI (+)in tumor site, as shown in Table 3. The correlation test showed that the tumor site and EL positive had significant correlation(P < 0.05), and the right colon of EL positive rate was lower than the left colon.

## Discussion

PEL comprised a relatively delicate layer of elastic fibers that laid just deep to the mesothelium. Therefore, PEL might be as a marker of CRC cancer of serosal invasion [8]. The importance of the PEL in pathological conditions such as neoplasia was that it could provide a surrogate anatomical marker in those cases where tumor destruction or prominent fibro-inflammatory changes had distorted and effaced the native serosa [8].

In our study, there were 47 (EL (-), 18.5%) cases that could not be identified EL using elastic fibers stain. In pathological situation, many reasons lead to the elastic lamina change in shape of size, structure and position, these partly were likely to lead to pT3 cases EL (-). The normal peritoneal surface was not smooth and flat but followed the undulating contour of the pericolic fat with clefts. Identification of these serosal clefts could be difficult, especially in the pathological setting, since they may be distorted or damaged by adjacent tumor infiltration or by inflammatory and fibrotic changes [9, 10]. CRC frequently elicited desmoplastic changes that included fibroblasts/ myofibroblasts, inflammatory and immune cells, and so on. These non-neoplastic elements were increasingly recognized to play an important role in cancer progression [11, 12]. Even if there was no direct invasion of the tumor, these reactive stromal components could also damage normal anatomic structures including the peritoneum [13]. Finally, the serosa may be damage during surgery, fixation, or post-operative handling. Therefore, in some cases it could be difficult to identify residual mesothelium overlying a tumor [8].

In Our study, the EL and ELI positive rate were 81.5% and 42.1% respectively. There is a wide variation in the reported incidence of EL positive and ELI positive, ranging from 41.4% - 98.2%, and 16.7–44.0% respectively [7, 14–18]. The existence of these problems had its special anatomy and histology of reasons. There were the possible reasons that led to this kind of difference. 1). the differences of the stage and tumor site might affect the results of study. 2) differences of sampling method: Ludeman L et research pointed out that the importance of sampling in the ELI diagnostic role, and pointed out that was not the most important part of the flat peritoneal mesothelium cell covered, but the area of peritoneal change direction, especially acute Angle area [10]. The cases of our study were based on "cross-shaped" sampling method, as much as possible sampling the deepest depth of tumor invasion. 3) The difference of blocks number for elastic fibers staining: Study shown that if taking more than 2 blocks for elastic lamina staining, EL positive rate and ELI positive rate were significantly improved, especially the ELI positive rate [8–10,19–20]. The average per patient of blocks we took was 3.24. Therefore, we recommend using three blocks for each case to take stain in order to improve the EL positive rate. In addition, if gross examination showed tumor near the surface of the serosa, but elastic fiber staining did not show the invasion of serosal surface, we should take serial sectioning, increase the number of blocks and repeated staining to rule out real serosal invasion [10]. 4) Difference of diagnostic criteria: The understanding of the diagnostic criteria of observer and the practical application might be different. But so far there were no the consistency of the research and solution.

T4 stage was the important risk factor that NCCN guidelines pointed out should be taken chemotherapy in CRC patients. Because many patients are difficult to follow up, our study further analyzed the relationship of the ELI and adverse prognostic factors for colon cancer. The results showed that there were significant differences and the positive correlation in the lymph node metastasis, vein invasion and tumor buds between the pT3 ELI (-) and the pT3 ELI (+), pT3 ELI (-) and pT4a. However, there were no significant difference between the group of the pT3 ELI (+) and pT4a in the same factors. This may mean that these two groups of patients in pT3 ELI (+) and pT3 ELI (-), although in the same stage, might have different prognosis. Patients with pT3 ELI (+) might have similar prognosis to patients with pT4a and should be used the same treatment. We proposed, ELI might be the prognostic risk factors of colon cancer with IIstage and might be the indicator of postoperative adjuvant chemotherapy. This required us to continue to do more detailed research to confirm.

There were no significant differences in lymph node metastasis, nerve invasion and tumor bud between pT3 EL (-) and the pT3 ELI (-), and pT3 EL (-) and pT3ELI (+) have significant difference in same factors. This might be suggest that pT3 EL (-) might have consistency with the pT3 ELI (-) in poor prognosis factors in colon cancer, and have significant difference with the pT3 ELI (+). Through the follow-up study of the prognosis of 244 cases of stage T3N0MO colon cancer, Liang and his group found that ELI (+) group of patients with significantly lower of 5 years DFS (60%) and OS (66.7%) than ELI group (-) 5 years DFS (87.8%) and OS (92.7%) and EL (-) DFS 5 years (82.5%) and OS (86.0%), while in ELI (-) and EL (-) they did not seen similar findings [15]. our results had consistent with their study. Based on our results, we speculated that for patients with pT3 colon cancer, if there was no elastic lamina confirmed by adequate and carefully sampling and selecting blocks for staining, it was possible that the patient could be grouped to the pT3 ELI(-) and might be take the same therapy. Given the importance of serosal ELI on postoperative treatment, prognosis of patients and limited research on this group of patients, we suggest that this group of patients should be described separately in clinical work, and to develop appropriate treatment programs based on other prognostic factors of patients.

Our study would group the caecum to the right colon, descending colon and sigmoid colon are classified for the left colon. There was significant correlation between tumor site and EL positive in the pT3 stage with colon cancer, and the right half colon EL positive rate was lower than the left half colon. Grin’s results also showed the right-side colon EL positive rate was lower than those of left colon [7]. In the cecum and ascending colon parts due to relatively thin bowel wall, elastic plate structure was slim and difficult to identify [7]. At the same time, we thought the location of tumor might be another reason.

In conclusion, ELI might be the prognostic risk factors of colon cancer with II stage and might be the indicator of postoperative adjuvant chemotherapy. Patients with pT3 ELI (+) might have similar prognosis to patients with pT4a and should be used the same treatment. For patients with pT3 colon cancer, EL(-)might have similar prognosis as ELI (-)and might be take the same therapy. In addition, the right half colon EL positive rate was lower than the left colon. Elastic staining might be a useful tool to help determine the invasive depth and staging of colon cancer.
